# Single-cell sequencing reveals the functional heterogeneity of melanoma cells and their crosstalk with the tumor microenvironment

**DOI:** 10.3389/fgene.2026.1779213

**Published:** 2026-04-02

**Authors:** Jianxiong Qiao, Xiaxia Li, Zhen Qin, Peigang Zhao, Hanghang Zhou, Lihong Qiu, Yue Yuan, Lin Zhong

**Affiliations:** 1 Department of Plastic Surgery, The Second Hospital & Clinical Medical School, Lanzhou University, Lanzhou, Gansu, China; 2 Department of Anesthesiology, The Second Hospital & Clinical Medical School, Lanzhou University, Lanzhou, Gansu, China; 3 The School of Clinical Medicine, Gansu Medical College, Pingliang, Gansu, China; 4 The Second School of Clinical Medicine, Lanzhou University, Lanzhou, Gansu, China; 5 Department of Plastic surgery, Tianjin Stomatological Hospital, School of Medicine, Nankai University, Tianjin, China; 6 Department of Reproductive Medicine, Key Laboratory for Reproductive Medicine and Embryo of Gansu, The First School of Clinical Medicine, The First Hospital of Lanzhou University, Lanzhou, Gansu, China

**Keywords:** eIF5A, machine learning, melanoma, prognosis, ScRNA-seq, tumor immune microenvironment

## Abstract

**Background:**

Cutaneous melanoma is a highly aggressive malignancy characterized by significant heterogeneity, rapid progression, and variable treatment responses. Understanding the functional diversity of melanoma cells and their interactions with the tumor microenvironment (TME) is crucial for developing effective therapeutic strategies and identifying prognostic biomarkers.

**Methods:**

We performed comprehensive single-cell RNA sequencing (scRNA-seq) analysis of 70,760 cells from 11 melanoma samples. Data processing was conducted using Seurat v4.3.0 with Harmony integration. Cell-cell communication was inferred using CellChat, and pseudotime trajectory analysis was performed using Monocle 2. A prognostic model was constructed by integrating 10 machine learning algorithms within a leave-one-out cross-validation (LOOCV) framework using the TCGA-SKCM cohort. Experimental validation was performed using immunofluorescence analysis on clinical specimens from seven melanoma patients.

**Results:**

We identified seven major cell types and characterized nine distinct melanoma cell subpopulations with unique molecular signatures. Notably, subpopulations Mela4, Mela6, and Mela9 demonstrated significant associations with favorable patient prognosis and exhibited the highest interaction strength with immune cells in the TME. Cell communication analysis revealed that these subpopulations primarily engaged in signaling through MIF-CD74/CD44/CXCR4 and MHC-I pathways, with CD8^+^ T cells being the predominant signal recipients. Pseudotime trajectory analysis identified critical genes (CYR61, JUN, RHOC) involved in melanoma cell state transitions. Using an integrative machine learning approach, we developed a melanoma cell-associated signature (MRS) comprising 15 genes that achieved a mean C-index of 0.675 across validation cohorts. Furthermore, High EIF5A expression was significantly associated with poor patient outcomes (p < 0.001), Immunofluorescence analysis showing significantly elevated EIF5A expression in melanoma tissues compared to controls (p < 0.01).

**Conclusion:**

This study reveals the functional heterogeneity of melanoma cells and their interactions with the immune microenvironment, identifies key subpopulations, prognostic signatures, and EIF5A as a plausible prognostic biomarker candidate and potential therapeutic target that warrants mechanistic validation in melanoma.

## 1 Introduction

Cutaneous melanoma arises from melanocytes and is recognized as a deadly tumour due to its high heterogeneity, aggressiveness, and rapid progression (Long et al., 2023). It presents a grave threat to human health. Although melanoma accounts for a relatively small proportion of all skin tumours, it is the primary cause of skin tumour-related death due to its high aggressiveness (Motwani and Eccles, 2021; Arnold et al., 2022). Current treatment regimens for advanced melanoma include immune checkpoint blockade therapy and BRAF/MEK inhibitor-based targeted therapies. Despite advancements in these approaches, the absence of reliable prognostic biomarkers to predict disease progression, combined with primary or secondary resistance, results in variable treatment outcomes (Atkins et al., 2023; Ziogas et al., 2021; Carlino et al., 2021; Kozar et al., 2019). Around half of the patients suffering from advanced melanoma eventually succumb to their disease (Long et al., 2023). This underscores the urgent need for more precise molecular biomarkers that have prognostic value and can guide treatment decision making.

Melanoma cells originate from neural crest-derived melanocytes, which are dispersed throughout the skin, eyes, and various other tissues during early development (Mort et al., 2015). The disease manifests as a spectrum of lesions, from benign melanocytic nevi to malignant melanoma, and melanocytes in different bodily locations are capable of developing into various subtypes of melanoma (Bastian, 2014). The pathogenesis of primary melanoma may be linked to multiple precursor lesions, and it features a complex array of mutations that disrupt crucial signalling pathways governing cell proliferation (such as mutations in BRAF, NRAS, and NF1), growth and metabolism (such as mutations in PTEN and KIT), and apoptosis resistance (such as mutations in TP53) (Hayward et al., 2017). Tumour invasion and metastasis, which are significant drivers of disease deterioration, are shaped by a multitude of factors and signalling pathways within the tumour microenvironment (Motwani and Eccles, 2021). While the immune microenvironment is pivotal in these processes, the specific immune cells and genes involved in melanoma metastasis remain poorly understood. A thorough examination of melanoma cell states, functions, and interactions with the immune microenvironment will aid in the identification of targets for treatment, and the application of such strategies may improve the prognosis of melanoma patients.

Single-cell RNA sequencing (scRNA-seq) has greatly advanced our understanding of cellular heterogeneity in a variety of pathological tissues in recent years (Jiang et al., 2022; Jovic et al., 2022). This high-throughput analytical technique has delineated the transcriptomic characteristics of melanoma across various grades and molecular classifications (Tirosh et al., 2016; Thrane et al., 2018). In our study, we methodically categorized melanoma cells and elucidated their molecular and biological attributes. Moreover, we explored the prognostic significance of characteristic genes in melanoma cell-associated subtypes using a machine learning approach, offering valuable insights for disease prognosis evaluation and the development of personalized treatment strategies.

## 2 Materials and methods

### 2.1 Data collection

The scRNA-seq dataset comprises 70,760 cells from 11 samples, sourced from the Gene Expression Omnibus with accession number GSE215120 (Zhang et al., 2022). This dataset includes 45,532 cells derived from acral melanoma (AM) and 25,228 from cutaneous melanoma (CM), processed using the HiSeq X Ten platform (*Homo sapiens*). Additionally, all bulk RNA-seq data were obtained from The Cancer Genome Atlas (TCGA) database (https://portal.gdc.cancer.gov/).

### 2.2 Data processing

Single-cell RNA-seq data were processed in R using Seurat v4.3.0. For each sample, raw counts were imported and Seurat objects were created using CreateSeuratObject (min.cells = 3, min.features = 40). Mitochondrial content was calculated with PercentageFeatureSet.

#### 2.2.1 Normalization, integration and clustering

Data were normalized using Seurat’s NormalizeData with LogNormalize (scale factor 10,000), highly variable genes were identified using FindVariableFeatures (selection.method = “vst”, nfeatures = 2,000), and expression values were scaled with ScaleData. Dimensionality reduction was performed by PCA using variable features. After merging all samples, batch integration was performed using Harmony v1.2.3 via RunHarmony, with “orig.ident” specified as the batch variable. Graph-based clustering was conducted on the Harmony embedding using FindNeighbors (reduction = “harmony”, dims = 1:40) followed by FindClusters (resolution = 0.8). UMAP and t-SNE visualizations were generated with RunUMAP and RunTSNE using the Harmony reduction (dims = 1:40).

#### 2.2.2 Doublet detection and removal

Potential doublets were identified using DoubletFinder v2.0.3. For each sample, DoubletFinder was run with PCs = 1:20, pN = 0.25, pK = 0.09, and an expected doublet rate of ∼8%; the expected number of doublets was set as nExp = round (0.08×N), where N is the number of cells after QC filtering. Cells classified as “Doublet” were removed prior to downstream analyses.

### 2.3 Cell type identification

We carried out differential expression analysis of genes within cell clusters using Seurat’s FindAllMarkers function to pinpoint marker genes for each cluster. The criteria for identifying marker genes included an adjusted P value <0.05, percent expression >0.25, and |log2 [fold change (FC)] | > 0.5. Following this, cell clusters were identified and annotated using the SingleR package, which assessed the composition patterns of marker genes. These annotations were then manually verified and corrected with the aid of the CellMarker database. Malignant cells were annotated based on their correlation with the data provided by the cell annotations.

### 2.4 Survival analysis of melanoma signatures in TCGA-SKCM

We evaluated the association between each melanoma subpopulation signature and overall survival (OS) in the TCGA-SKCM cohort. For each subpopulation, the signature gene set was derived from the top 10 highly expressed genes of that subtype. For each signature, a multivariable Cox proportional hazards model was fitted using the expression of the signature genes to calculate a risk score for each patient. An optimal cutpoint for the risk score was then determined using maximally selected rank statistics to stratify patients into High-Risk and Low-Risk groups. Kaplan–Meier curves were generated, and group differences were assessed using the log-rank test. To account for multiple comparisons across signatures, P values were adjusted using the Benjamini–Hochberg procedure to control the false discovery rate (FDR), and both nominal P values and FDR-adjusted values are reported.

### 2.5 Cell communication

The “CellChat” R package v1.1.3 was utilized to infer cell communication within the tumor microenvironment, based on receptor-ligand interactions (Jin et al., 2021). We counted the number of links and gathered communication probabilities to construct the communication network. The number of interactions and the total strength of interaction between two arbitrary cell populations are visually displayed. Scatterplots are created to illustrate the main sender (signal source) and receiver cells (target) in two-dimensional space, to identify major signal senders and receivers among cell populations. Additionally, we employed a pattern recognition approach, the global communication model, to analyze how multiple immune cell types and signaling pathways interact.

### 2.6 Construction of the prognostic signature using machine learning

We utilized melanoma cell-related signatures (MRS) for a series of validations to ensure accuracy, stability, and reproducibility. We integrated up to 10 machine learning algorithms, including Random Survival Forest (RSF), Elastic Net (Enet), Lasso, Ridge, Stepwise Cox, CoxBoost, Cox Partial Least Squares Regression (plsRcox), Supervised Principal Component (SuperPC), Generalized Boosting Regression Model (GBM), and Survival Support Vector Machine (survival-SVM). We tested 101 algorithm combinations under the framework of leave-one-out cross-validation (LOOCV). Bulk RNA-seq data and corresponding overall survival (OS) information for melanoma were obtained from TCGA-SKCM.

#### 2.6.1 Data split

TCGA-SKCM patients were randomly split into a training set (60%) and an internal hold-out validation set (40%) using a fixed random seed. The validation set was not accessed during feature selection, algorithm selection, or hyperparameter tuning.

#### 2.6.2 Input features and preprocessing

Candidate genes were defined *a priori* (122 candidates derived from the intersection of subgroup-specific marker genes and pseudotime-varying genes). Expression matrices were organized with samples as rows and genes as columns. Z-score standardization (centering and scaling) was performed separately for the training set, and for the validation set within each cohort level.

#### 2.6.3 Model training and comparison in the training set

We evaluated different algorithms and combinations. For each combination, a pre-selection step (e.g., Stepwise Cox, Elastic Net, RSF-based selection) was performed using the training set only to identify a reduced feature set, followed by fitting the final survival model on the training set using the selected genes. For algorithms requiring hyperparameter tuning (e.g., Elastic Net, CoxBoost, GBM), tuning was conducted within the training set using internal K-fold cross-validation as implemented in the corresponding packages (e.g., cv.glmnet). Models retaining fewer than 5 genes were excluded.

#### 2.6.4 Risk score calculation and evaluation

For each trained model, a risk score was computed as the model linear predictor. Each model was evaluated in the internal validation set using the concordance index (C-index), calculated from a Cox model of OS on the predicted risk score. The final 15-gene MRS corresponded to the selected best-performing model, and its gene set and coefficients were used for downstream analyses (Kaplan–Meier survival, time-dependent ROC/AUC, and hazard ratios based on Cox regression).

#### 2.6.5 Evaluation in the internal validation set

For each candidate model trained on the 60% training set, we computed risk scores in the 40% internal validation set and quantified predictive performance using the concordance index (C-index). Time-dependent ROC/AUC, Kaplan–Meier curves, and hazard ratios from Cox regression were then reported for the selected best-performing model.

### 2.7 Pseudo-time trajectory analysis

Pseudo-time trajectories were inferred using Monocle 2 on the melanoma cells of interest (Qiu et al., 2017). Raw UMI counts from the Seurat object were used to construct a CellDataSet with a negative binomial expression model (negbinomial.size ()), followed by size-factor and dispersion estimation. Ordering genes were selected as highly variable genes based on Monocle’s dispersion model, retaining genes with mean_expression ≥0.1 and dispersion_empirical ≥ dispersion_fit, and these genes were set with setOrderingFilter. Cells were embedded using DDRTree (reduceDimension, max_components = 2) and ordered along the learned principal graph using orderCells. Given that melanoma cells often exhibit heterogeneous and potentially branching state transitions, we interpreted the DDRTree trajectory as a low-dimensional state-transition manifold inferred from transcriptional similarity rather than as evidence of a single, deterministic lineage. Gene dynamics along pseudotime were visualized using plot_pseudotime_heatmap.

### 2.8 Copy-number variation inference

Copy-number variation (CNV) profiles were inferred using inferCNV from raw UMI count matrices extracted from the Seurat object. Gene genomic coordinates were provided via a curated gene-order file derived from human gene position annotations. Cell-type labels were taken from the Seurat metadata and supplied as the inferCNV annotation file. inferCNV objects were constructed using the average expression across all cells as the reference baseline. CNV inference was performed using infercnv::run with cutoff (0.1), group-aware clustering (Ward.D2 hierarchical clustering), and denoising enabled, while HMM-based calling was not applied. Given the lack of a clearly defined normal reference group, we used “all cells” as a pragmatic reference; however, this choice may dilute the tumor–normal contrast.

### 2.9 Co-expression network analysis

Gene co-expression networks for the melanoma subclusters Mela4/Mela6/Mela9 were constructed using hdWGCNA. Genes were included if detected in at least 5% of cells. To improve robustness given modest cell numbers per subcluster, we applied hdWGCNA’s metacell strategy, aggregating cells by melanoma subclusters (KNN on the Harmony embedding; k = 25, max_shared = 10). Networks were built from the log-normalized expression matrix (RNA data slot). A signed network was used, and the soft-thresholding power was selected by TestSoftPowers; the final networks were constructed using soft_power = 7. For network inference, we used metacell-aggregated expression and SCT variable features (variable.features.n = 2000). The expression matrix used for network construction comprised 2,151 metacells × 2000 genes. Using these inputs, we identified six non-grey modules (M1–M6) (grey denotes unassigned genes). Module sizes (genes per module) were M1: 141, M2: 261, M3: 767, M4: 83, and M5: 104 genes. Module eigengenes were computed and harmonized across samples (group.by.vars = “group”), and intramodular connectivity (kME) was calculated for the corresponding group.

### 2.10 Multiple testing correction

For single-cell differential expression analyses performed with Seurat (FindAllMarkers/FindMarkers, Wilcoxon rank-sum test), p-values were adjusted for multiple testing using the Benjamini–Hochberg procedure (BH) within each comparison across all tested genes, and genes with FDR <0.05 were considered significant. For pathway enrichment analyses (GO/KEGG) and gene set analyses, p-values were adjusted using BH-FDR across all tested gene sets; terms with FDR <0.05 were reported. For comparisons involving multiple melanoma subpopulation signature scores in survival analyses, the resulting log-rank p-values were adjusted using BH-FDR across the tested subpopulations. Unless otherwise stated, all reported “adjusted p-values” refer to BH-FDR.

### 2.11 Clinical patient characteristics and immunoffuorescence staining

This study collected data from patients hospitalized in the Department of Plastic Surgery, the Second Hospital of Lanzhou University, between January 2024 and June 2025. Seven patients diagnosed with cutaneous malignant melanoma were enrolled, including 5 females (71.4%) and 2 males (28.6%), with a median age of 68 years (range: 42–75 years). Adjacent non-tumor tissues from the same patients served as controls. All patients had no history of other malignant tumors. Informed consent was obtained from all participants in accordance with the Declaration of Helsinki. This study was approved by the Medical Ethics Committee of the Second Hospital of Lanzhou University (Approval No. 2025A-1349).

The tissue specimens were fixed in 4% paraformaldehyde, embedded in paraffin, and cut into 5 μm thick sections. After deparaffinization, antigen retrieval, and blocking for 1 h, the sections were incubated with the primary antibody against EIF5A (1:300, cat# DF6754, Affinity, China) overnight at 4 °C. On the following day, the sections were incubated with the Goat Anti-Rabbit IgG secondary antibody (1:300, cat# SA00013-2, Proteintech, China) at room temperature and counterstained with DAPI. Images were finally captured using a fluorescence microscope.

### 2.12 Statistical analysis

All statistical analyses and data visualizations were conducted using R software (version 4.1.3). The Pearson correlation coefficient was used to assess the correlation between two continuous variables. For quantitative data, values between subgroups were compared using a two-tailed, unpaired Student’s t-test or one-way analysis of variance (ANOVA) with Tukey’s multiple comparisons test. A p-value of less than 0.05 was considered statistically significant.

## 3 Results

### 3.1 Single-cell sequencing and cell distribution analysis of AM and CM tissues

**FIGURE 1:**
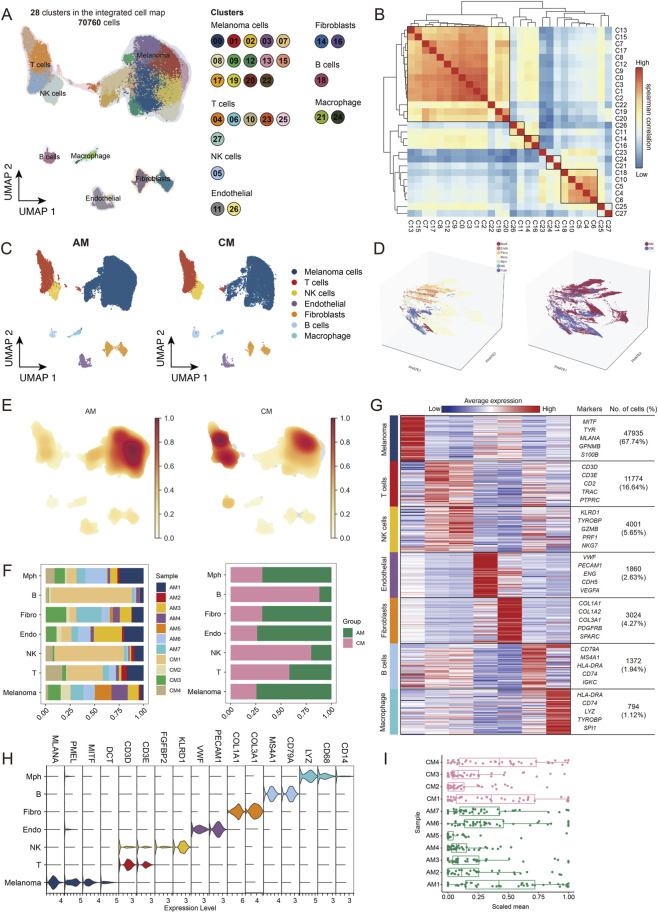
Single-cell characterization of the AM and CM ecosystem. **(A)** Visualization of single-cell RNA-seq data by UMAP of 70,760 cells from GSE215120. The above cells were divided into 28 clusters and 7 cell types. **(B)** Heatmap depicting pairwise correlations among 28 clusters derived from 11 samples. **(C)** UMAP plots showing the distribution of cells in AM and CM groups, coloured by cell type. **(D)** The PHATE dimensionality reduction method shows the distribution of cells in a three-dimensional coordinate system, colored by cell type and group. **(E)** Density plots show the relative abundance of cells in different groups. **(F)** Stacked bar graph showing the proportion of sample distribution, proportion of sample type, and number of cells for each cell type. **(G)** Heatmap showing the specificity of the cell type annotations, with the names of the different cell types labelled on the left. **(H)** Violin plots showing the expression of marker genes in each cell type. **(I)** Inflammation levels of individual samples.

In this study, single-cell sequencing analysis was performed on 11 samples from the GSE215120 dataset. We applied strict quality control measures to our samples, including the removal of low-quality cells and the elimination of potential doublets (Supplementary Figures S1A,B). Following these procedures, we retained a total of 70,760 cells, comprising 45,532 cells derived from AM tissue and 25,228 from CM tissue. We analysed 25,834 genes in the scRNA-seq validation dataset. Using the “Harmony” method, we integrated samples from different origins to eliminate batch effects and conducted normalization, scaling, and PCA dimensionality reduction, preserving the first 20 principal components. These cells were then clustered and visualized using the UMAP method (Figure 1A). We organized the cells into 28 clusters and further categorized them into 7 cell lineages based on well-documented cell type-specific genes (Zhang et al., 2022) and singleR results: melanoma cells highly expressing MLANA and PMEL, T cells expressing CD3D and CD3E, NK cells with KLRD1, endothelial cells marked by VWF and PECAM1, fibroblasts with COL1A1 and COL3A1, B cells with CD79A and MS4A1, and monocytes/macrophages expressing LYZ and CD68. The consistency of cell types between clusters was also verified (Figures 1B,H). These markers have also been utilized to differentiate various cell types within melanoma (Zhang et al., 2022). Figure 1C shows the distribution of various cell types in the AM and CM groups. PHATE three-dimensional visualization captured local and global non-linear structures, revealing structural differences between AM and CM that complemented the UMAP analysis (Figure 1D). The density plot and stacked bar chart indicate that the relative abundance of NK and T cells in the CM group is higher than in the AM group (Figures 1E,F). Figure1G highlight the top 50 genes with specific high expression in each cell type, indicating both the reliability and specificity of the cell annotations. Notably, genes associated with inflammatory responses were significantly overexpressed in samples AM1, CM1, and CM4 compared to other samples (Figure 1I). This finding is consistent with the higher proportions of T cells, NK cells, and B cells observed in these samples, suggesting an activated immune microenvironment.

### 3.2 Different subtypes of melanoma cells have unique functional characteristics

**FIGURE 2:**
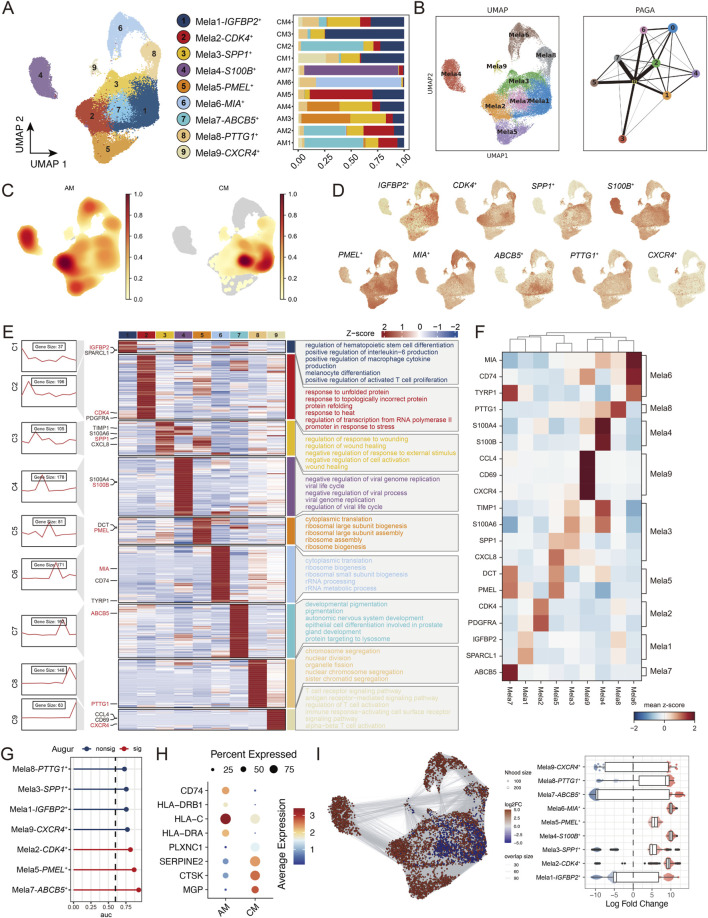
Melanoma cell subtypes exhibit distinct functional profiles. **(A)** UMAP plot demonstrating melanoma cell segmentation results, showing cell types by colour. Stacked bar graph showing the proportion of cells in each sample. **(B)** PAGA algorithm shows the trajectory of each subgroup. **(C)** Density plots show the relative abundance of cells in different groups. **(D)** Expression of representative genes in each melanoma cell subtype. **(E)** Left: This series of graphs illustrates the dynamic pattern of changes in representative differentially expressed genes (DEGs) in each melanoma cell population. Middle panel: Heatmap showing representative DEGs between each cell population. Right panel: representative enriched gene ontology (GO) terms for each cluster. **(F)** Heatmap showing expression of selected genes in each melanoma cell subtype. **(G)** Random forest-based Augur classifier identifies subpopulations with the greatest transcriptome perturbations between AM and CM. **(H)** Bubble chart showing AM and CM specific expression genes. **(I)** Embedding the Milo k-nearest neighbour differential abundance testing results for melanoma cells.

As a critical component of tumours, melanoma cells are known to regulate tumour formation, metastasis and invasion and affect patient prognosis (Balch et al., 2009; Guerry et al., 1993; Shain and Bastian, 2016), drawing our attention to their prevalence in the dataset (Figure 1A). UMAP visualization revealed 9 transcriptionally distinct melanoma cell subpopulations (Figure 2A, left). The composition of these subpopulations varied substantially across samples (Figure 2A, right), indicating significant inter-tumor heterogeneity. To facilitate biological interpretation and cross-study comparability, we further mapped these subpopulations onto the tumor-intrinsic programs defined by Zhang et al. (Supplementary Figure S2). Next, PAGA analysis revealed the connectivity among melanoma subpopulations (Figure 2B). Mela1, Mela3, and Mela7 showed strong interconnections, suggesting potential state transitions, while Mela4 remained largely isolated from other subpopulations. The density plot indicates that Melanoma4, Melanoma5, and Melanoma6 seem to be more frequently observed in AM, while Melanoma1 and Melanoma7 are more likely to be observed in CM (Figure 2C). To accurately characterize the gene expression patterns across cellular subpopulations, we assigned scores to genes within each individual cell based on their relative expression levels. Using these scores, we applied an unsupervised clustering method to distinguish unique gene expression profiles within each subtype (Figures 2D–F). Melanoma2 cells exhibited high expression of CDK4, a key regulator of the cell cycle critical for the G1/S phase transition (Spring et al., 2020), and aberrant CDK4 activation is implicated in cell cycle dysregulation across several cancers (Zhang et al., 2018; Voigt and Quelle, 2023; Padhye et al., 2021). Melanoma7 cells showed elevated expression of the tumour stem cell marker ABCB5, which is suggested to confer resistance to 5-fluorouracil in tumour cells (Ksander et al., 2014; Kugimiya et al., 2015). Melanoma9 cells overexpressed CXCR4, a G protein-coupled receptor involved in cell migration and metastasis in various cancers (Burger and Kipps, 2006). Furthermore, we noted high expression levels of key molecules in certain cell clusters, such as SPP1, which play significant roles in regulating cell adhesion, migration, and the immune response, in Melanoma3 cells (Kahles et al., 2014). Interestingly, in the TCGA-SKCM bulk RNA-seq data, high CDK4 and PMEL expression were correlated with poorer patient outcomes than low CDK4 and PMEL expression (Supplementary Figures S3A,B). These findings indicate distinct gene expression patterns across different melanoma subpopulations, which may affect melanoma cell behaviour and the response to therapy.

Augur allows us to determine the cell types experiencing the largest transcriptomic perturbations based on a random forest classifier. The results indicate that Mela7-ABCB5+ underwent the most significant changes (Figure 2G). We analyzed the genes that changed in Mela7 and found that in AM, Mela7 highly expresses HLA-DRA, HLA-C, HLA-DRB1, and CD74, suggesting that acral melanoma may have a stronger antigen presentation ability. In contrast, the upregulation of MGP, CTSK, SERPINE2, and PLXNC1 in CM suggests that melanoma may have higher invasiveness and metastatic potential (Figure 2H). We used the k-nearest neighbor statistical method for differential abundance analysis, and the results show that compared to CM, the AM group exhibited upregulation in the proportions of multiple types, such as Mela2-6 (Figure 2I).

### 3.3 Identification of functional melanoma cell subpopulations associated with patient prognosis

**FIGURE 3:**
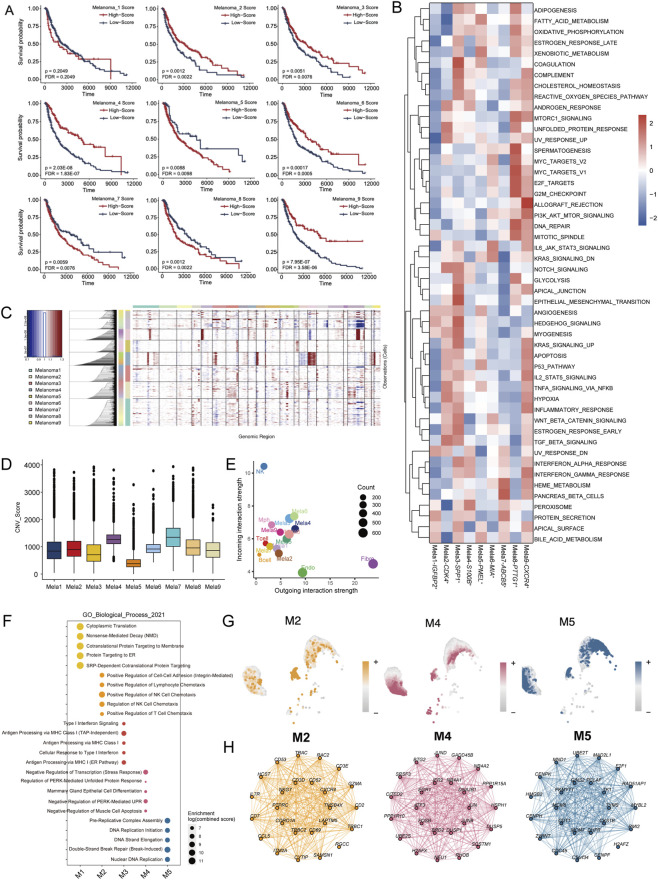
Delineating prognostic melanoma cell subsets based on functional characteristics. **(A)** Kaplan‒Meier survival analysis of the top 10 most highly expressed genes for each subgroup of melanoma cells in the TCGA-SKCM cohort. **(B)** GSEA heatmap of the 50-marker gene set in the MSigDB database with melanoma cells shown by subgroup. **(C)** Heatmap showing the CNV profile of each melanoma cell cluster. Red and blue represent high and low CNV levels, respectively. **(D)** Box plot showing the relative CNV scores of each subtype. **(E)** Scatter plot showing the incoming and outgoing interaction strengths of various cell types, including melanoma subtypes and immune cells. **(F)** GO enrichment analysis of each hdWGCNA module. **(G)** UMAP plots of hdWGCNA-identified co-expression modules 2, 4 and 5. **(H)** hdWGCNA module network plots showing the top 25 genes by the eigengene-based connectivity for module 2, 4, 5. Each edge represents the co-expression relationship between two genes in the network.

To evaluate the clinical impact of each melanoma cell subpopulation, we utilized an independent SKCM cohort from TCGA. Kaplan-Meier survival analysis revealed distinct prognostic associations among melanoma cell clusters (Figure 3A). Higher signature scores of clusters 2, 3, 4, 6, and 9 were significantly associated with better overall survival (OS), whereas higher scores of clusters 5, 7, and 8 correlated with worse OS (log-rank test, all p < 0.01). Cluster 1 showed no significant prognostic value (p = 0.2) (Supplementary Table 1). Time-dependent ROC curves for each cluster’s signature genes in predicting patient OS are presented in Supplementary Figures S4B–J, with AUC values reported at 1, 3, 5, 7, and 10 years, further corroborating the prognostic relevance of these subpopulation-specific transcriptional programs. Of note, the pairwise correlation structure among subpopulation signature scores (Supplementary Figure S4A) revealed moderate inter-cluster associations despite the absence of gene overlap between clusters, suggesting that at the bulk transcriptomic level, distinct subpopulation signatures may partly capture shared upstream transcriptional programs rather than fully orthogonal biological states. These findings align with prior research suggesting that different functional cell subtypes exhibit distinct biological behaviours and lead to different treatment responses, influencing patient prognosis (Zhang et al., 2022). Using MSigDB hallmark gene sets, we scored each melanoma cell subtype and employed the limma algorithm for differential expression analysis (Figure 3B). InferCNV analysis revealed heterogeneous copy number alterations across melanoma clusters (Figure 3C). Clusters 4 exhibited prominent copy number amplifications in certain genomic regions, while cluster 7 displayed both amplifications and deletions with the highest overall CNV burden (Figure 3D).

We used CellChat to analyze the interactions between various melanoma subtypes and other cell types. Interestingly, among melanoma cell clusters, Mela4, Mela6, and Mela9, which are associated with better patient survival, exhibited the highest interaction strength with other cell types in the tumor microenvironment (Figure 3E). We used high-dimensional weighted gene co-expression network analysis (hdWGCNA) to define five gene co-expression modules in Mela4, Mela6, and Mela9, and constructed a cell type-specific gene interaction network for each module. Notably, M2, M4, and M5 were common across these three melanoma subgroups. Among them, co-expression module M2 is involved in immune cell chemotaxis, with hub genes including CD3D, CD3E, CD2, CD7, and CXCR4. Co-expression module M4 is involved in oxidative and endoplasmic reticulum stress, while co-expression module M5 is involved in DNA replication and repair-related pathways (Figures 3F–H).

### 3.4 Directed immune signalling occurs between different melanoma cell subpopulations

**FIGURE 4:**
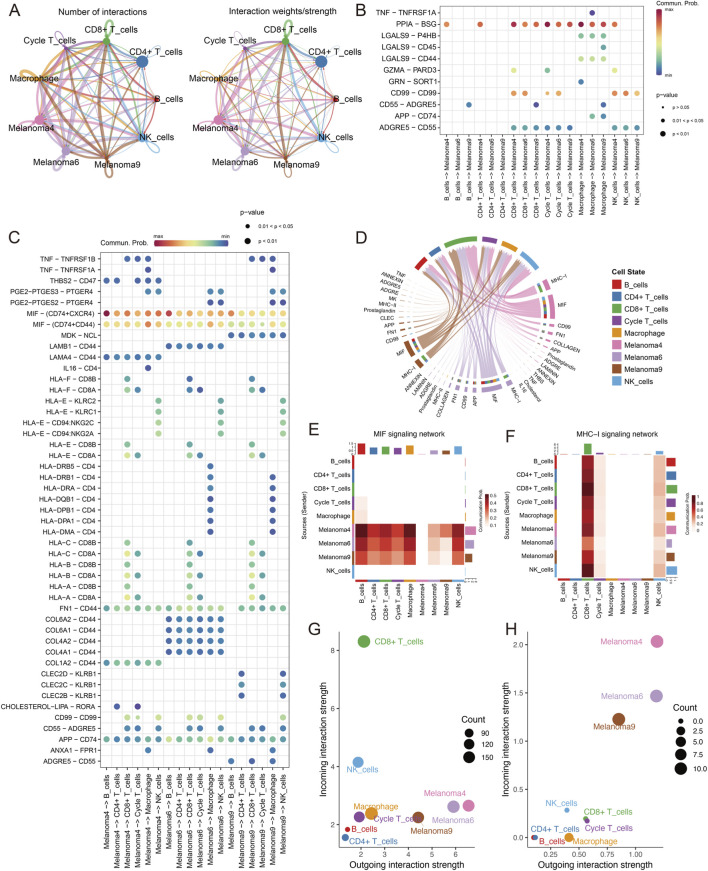
Cell communication analysis in melanoma subpopulations. **(A)** Circle plot showing the number (left) and strength (right) of interactions between melanoma cells and immune cells. **(B,C)** Bubble plots showing interactions between overexpressed ligands and receptors. The bubble size represents the P value generated by the alignment test, and the colour represents the likelihood of interaction, with the melanoma cell subgroups as signal receivers in the B plot and signal emitters in the C plot. **(D)** Circle plots visualizing the receptor‒ligand interactions of melanoma cell subgroups on various immune cells. **(E,F)** Heatmaps showing the interactions of various cell types with the MIF **(E)** and MHC-I **(F)** signalling pathways. **(G,H)** Dot plots showing the major senders and receivers. The X- and Y-axes are the total efferent or afferent communication probabilities associated with each group, respectively. The size of the dots is positively correlated with the number of inferred links (efferent and afferent) associated with each cell cluster. The colours of the dots represent different cell groups.

Guided by the prognostic insights from Figure 3A, we focused on the 3 cell subgroups (Mela4, Mela6, and Mela9) most significantly associated with patient OS. Using the CellChat tool, we simulated ligand‒receptor interactions between various cell types, constructing networks of intercellular communication. Subgroup 4 emerged as the predominant contributor to both incoming and outgoing signals within the immune signalling network (Figure 4A; Supplementary Figures S5A–C). We analysed the interactions in which melanoma cell subgroups acted as signal receivers and immune cell subpopulations acted as afferents. As recipients, the melanoma cell subgroups predominantly received inferred signals via the PPIA–BSG pathway from immune cells (Figure 4B). Conversely, all three subgroups acted as signal senders, showing robust inferred MIF-related communications toward immune cell receptors CD74, CD44, and CXCR4 (Figure 4C). Importantly, a substantial proportion of inferred outgoing communications from melanoma cell subgroups were directed toward CD8^+^ T cells and NK cells (Figure 4D). The inferred MIF-related communications from these subgroups targeted an array of immune cells, including B cells, CD4^+^ T cells, CD8^+^ T cells, circulating T cells, and NK cells (Figure 4E). MIF was recognized as a key factor in both local and systemic inflammation that might affect tumour immune evasion (Wen et al., 2022). Additionally, these melanoma cell subsets showed pronounced MHC-I–associated inferred communications toward CD8 +T cells (Figure 4F), which may be consistent with increased antigen-processing/presentation programs and/or interferon-driven immune interactions (Wu et al., 2023). Overall, CD8^+^ T cells stood out as the primary signal recipients (Figure 4G), with subgroup 4 cells identified as the primary signal origin (Figure 4H). Considering all the ligand‒receptor interactions within the MIF and MHC-I pathways, subgroup 4 was the main contributor, both emitting and receiving signals. Notably, the inferred MIF- and MHC-I–mediated interactions were not functionally validated at the protein level in this study and should be regarded as hypothesis-generating.

### 3.5 Key events in the altered proposed temporal trajectories of melanoma cells

**FIGURE 5:**
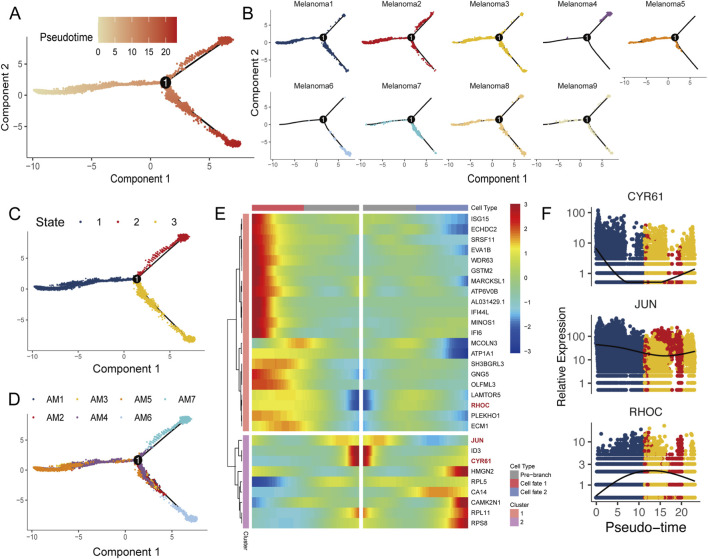
Trajectory analysis of melanoma populations. **(A–D)** Monocle analysis for melanoma cell subgroup trajectory inference, coloured by **(A)** proposed time, **(B)** subgroup, **(C)** inferred developmental state, and **(D)** sample source. **(E)** Pseudotemporal heatmap showing changes in the top genes before and after nodes were analysed by BEAM. **(F)** CYR61, JUN, and RHOC are differently coloured to represent inferred developmental states as pseudotime progresses.

To analyse shifts in melanoma cell states, we utilized the Monocle package to perform pseudotime trajectory analysis of nine melanoma cell subtypes, as shown in Figures 5A–C. We identified three distinct cellular states (Figure 5C) and discovered that cell subgroups 4 and 6 appeared at distinct positions of the trajectory, with subgroup 4 located at one endpoint (State 2) and subgroup 6 concentrated at the origin (State 1), potentially representing divergent cell fates within melanoma (Figure 5B). When the analysed samples were labelled, it was particularly noteworthy that melanoma cells from samples AM6 and AM7 were situated at both ends of the trajectory (Figure 5D). By employing BEAM analysis, we tracked significant gene changes across the trajectory before and after node 1 (Figure 5E; Supplementary Figure S6A), and within the top 30 genes, we pinpointed proteins associated with the extracellular matrix, such as CYR61, JUN, and the Rho GTPase family member RHOC (Figure 5F). CYR61 is known to trigger various signalling pathways by interacting with integrins, affecting processes such as cell proliferation, migration, adhesion, survival, and various cancer-associated processes including tumor growth and the regulation of anticancer drug resistance (Lau, 2011; Ascenção et al., 2022). JUN, part of the AP-1 complex, plays a role in cell proliferation, differentiation, and apoptosis, and the AP-1 pathway is implicated in the progression of various tumour types, including melanoma (Kappelmann et al., 2014). Moreover, RHOC reportedly facilitates the metastasis of multiple cancers, such as those of the breast, pancreas, and lung (Gua et al., 2018; Thomas et al., 2019).

### 3.6 Melanoma cell-associated features predict patient survival

**FIGURE 6:**
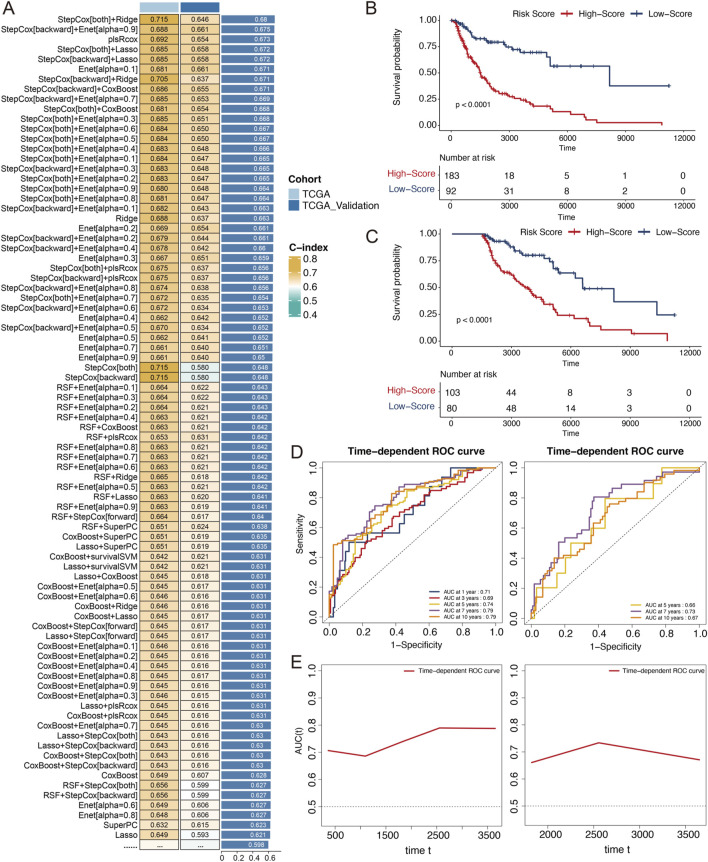
Establishment and validations of prognostic model via the machine learning-based integrative procedure. **(A)** Consensus MRS built and validated via an integrated machine learning-based procedure. A total of 101 prognostic models were constructed using the LOOCV framework, and the C-index of each model in all the validation datasets was further calculated. **(B)** K‒M survival curves of the MRS prognostic models in the training set. **(C)** K‒M survival curves of the MRS prognostic models in the validation set. **(D)** ROC curves assessing model performance. Left: 1-, 3-, 5-, 7-, and 10-year AUCs in the training set; Right: 5-, 7-, and 10-year AUCs in the validation set. **(E)** Sequential ROC curves to assess model performance.

Considering the significance of cell subgroups 4 and 6 in the monocle analysis and their impact on prognosis, we intersected genes that were highly expressed in these subgroups with those that showed significant variance during monocle trajectory progression, resulting in a set of 122 genes. Using the TCGA-SKCM dataset, we fitted these genes into 101 predictive models within the LOOCV framework. We calculated the C-index for each model across the testing and validation cohorts. The optimal model combination was StepCox [both]+Ridge, which had the highest average C-index of 0.68, although it utilized 45 features for survival prediction. The runner-up, StepCox [backward]+Enet [alpha = 0.9], used only 15 features and achieved an average C-index of 0.675 (Figure 6A). We employed StepCox [backward]+Enet [alpha = 0.9] to develop a melanoma cell-associated signatures (MRS) and verified the expression of the genes in the signature with single-cell data (Supplementary Figure S6B). In the TCGA-SKCM training and validation sets, the low-risk group exhibited significantly prolonged overall survival (OS, p < 0.0001, Figures 6B,C). The MRS effectively predicted 1-, 3-, and 5-year OS with area under the curve (AUC) values that indicated satisfactory specificity and sensitivity (Figure 6D, 1-year AUC = 0.71,3-year AUC = 0.69,5-year AUC = 0.74). Due to the smaller sample size in the validation set, we assessed the AUC for longer terms; we found a 5-year AUC of 0.66, a 7-year AUC of 0.73, and a 10-year AUC of 0.67 (Figure 6D). The consistent AUC trends underscore the model’s stability and reliability (Figure 6E). To assess clinical independence, calibration, and robustness, we further performed multivariable Cox regression with clinicopathologic covariates, calibration analyses, and gene-level ablation tests (Supplementary Figure S7). In addition, we compared the prognostic performance of the MRS with previously published melanoma prognostic signatures in the same cohort under a unified evaluation framework (Supplementary Figure S8). We present the MRS as a robust candidate, hypothesis-generating signature, pending validation in independent cohorts before broader clinical translation.

### 3.7 EIF5A is involved in the immune response of melanoma cells and is associated with patient prognosis

**FIGURE 7:**
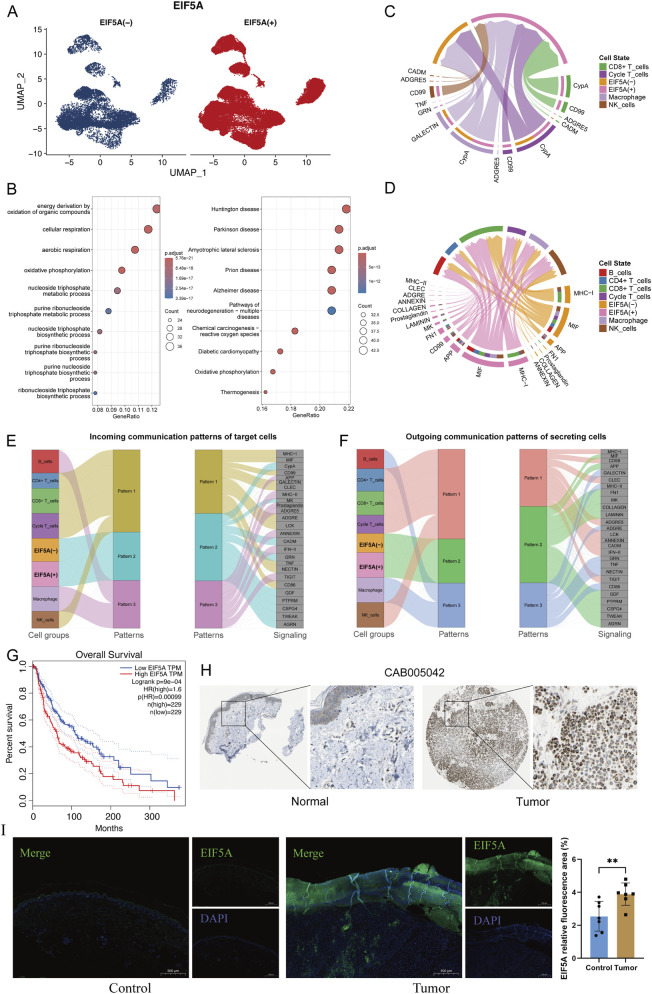
EIF5A is a potential therapeutic target for melanoma. **(A)** UMAP plot of melanoma cells coloured according to EIF5A expression. **(B)** Functional enrichment analysis of differentially expressed genes in the EIF5A (+) and EIF5A (−) groups for the top 10 GO and KEGG results, respectively. **(C,D)** Circle plots showing the interactions of the EIF5A (+) and EIF5A (−) groups with various immune cells as receivers and effectors, respectively. **(E,F)** River plots showing the communication patterns of the various cell groups and the output signalling pathways, as well as the communication patterns of the various cell groups and the input signalling pathways. **(G)** High expression of EIF5A in the TCGA-SKCM dataset was correlated with poor patient prognosis. **(H)** Based on the Human Protein Atlas database, EIF5A expression was found to be increased in melanoma tissue compared to normal skin tissue. **(I)** Representative immunofluorescence images of EIF5A in control and tumor groups (scale bar: 500 μm). Quantitative analysis of relative fluorescence area (n = 7). Data represent mean ± SD; **P < 0.01.

In our analysis, the EIF5A gene attracted our attention, standing out prominently among the genes associated with the Melanoma Risk Score (MRS). This gene is known to be highly expressed in various cancers, and its inhibitors hold potential for anticancer therapy (Sfakianos et al., 2022). However, the role of EIF5A in melanoma has not been well studied. We re-examined single-cell sequencing data of melanoma, categorizing cells into EIF5A-positive and EIF5A-negative groups based on expression (Figure 7A). We identified DEGs between these groups and performed GO and KEGG enrichment analyses. These analyses indicated that these genes are primarily involved in aerobic respiration and oxidative phosphorylation pathways, suggesting metabolic and nucleotide synthesis differences in the EIF5A-expressing melanoma cell subgroups (Figure 7B; Supplementary Figure S9). The EIF5A-positive group was found to receive a more complex array of immune cell signals than the EIF5A-negative group (Supplementary Figures S10A–C), with CD8^+^ T cells and NK cells signalling exclusively to the EIF5A-positive group (Figure 7C). Additionally, the EIF5A-positive group emitted more diverse signals to immune cells, with distinct pathways such as those involving CD99 and MK emerging (Figure 7D). The patterns of incoming and outgoing signals were specific to the cell type. Intriguingly, both the EIF5A-negative and EIF5A-positive groups showed parallelisms in their signal profiles (Figures 7E,F; Supplementary Figures S10D–F). Furthermore, high EIF5A expression in TCGA-SKCM bulk-seq data was significantly associated with worse patient survival (p < 0.001, Figure 7G), and the EIF5A protein level was notably greater in melanoma than in normal tissue according to the HPA database (Figure 7H). Immunofluorescence analysis showed that relative fluorescence area of EIF5A in the cutaneous malignant melanoma group was significantly higher than that in the control group (P < 0.01) (Figure 7I). Collectively, these findings support EIF5A as a candidate prognostic biomarker in melanoma and raise the possibility that EIF5A-related programs may be therapeutically actionable, which warrants further mechanistic studies and functional perturbation experiments in melanoma models.

## 4 Discussion

In this study, we conducted a comprehensive analysis of the GSE215120 datasets, in which we categorized these cells into seven major cell types spanning a diverse range of immune cells and melanoma cells. While several transcriptional patterns observed across Mela1–Mela9 are consistent with previously reported melanoma programs, our analysis extends these frameworks by systematically linking melanoma-cell states to TME crosstalk at single-cell resolution and downstream prognostic modelling. We identified nine melanoma cell subpopulations and noted distinct distribution patterns between acral and cutaneous melanoma samples, further characterizing the functional attributes of these subpopulations through gene expression analysis. For instance, Melanoma2 was found to express high levels of the cell cycle regulator CDK4, while Melanoma7 was marked by elevated expression of the tumour stem cell-associated gene ABCB5, which is potentially linked to tumour progression, drug resistance, and metastasis (Wang et al., 2017; Duvivier and Gillet, 2022). Using the independent TCGA-SKCM cohort, we assessed the impact of these melanoma cell subpopulations on patient outcomes. Our statistical analyses revealed significant correlations between the presence of certain subpopulations and overall survival (OS), suggesting that different melanoma cell subtypes may contribute to varying clinical prognoses. By examining inferred cell–cell communication, we found that melanoma subpopulations may engage in immune-related interactions with B cells, CD4^+^ T cells, and CD8^+^ T cells, with MIF-related signalling representing a prominent axis that has been linked to tumour immune escape (Noe and Mitchell, 2020). Furthermore, we observed pronounced MHC-I–associated inferred communications from specific subpopulations to CD8^+^ T cells. This pattern may reflect enhanced antigen-presentation machinery or interferon-driven adaptive responses; however, it could also be compatible with adaptive immune resistance and immune editing rather than uniformly beneficial anti-tumour immunity. Additionally, CellChat relies on curated ligand–receptor databases and expression-based probabilistic inference, and does not directly measure ligand availability, receptor engagement, downstream signalling activity, or immune effector function; Therefore, all inferred cell–cell communication results in this study require subsequent functional validation.

EIF5A, a critical translation initiation factor, is implicated in the development of various tumours. Research indicates that EIF5A may selectively enhance the translation of proteins essential for tumour cell proliferation (Fujimura et al., 2015; Dever et al., 2014; Gutierrez et al., 2013). The upregulation of EIF5A across different tumour tissues suggests its role in tumorigenesis (Mathews and Hershey, 2015). For instance, in pancreatic cancer, EIF5A has been shown to increase the expression of PEAK1, a downstream protein kinase. This activation cascade engages oncogenic factors such as YAP1/TAZ, fostering tumour growth and correlating with adverse outcomes (Fujimura et al., 2014). EIF5A activity hinges on hypusination, a unique posttranslational modification. Its tumour-promoting effects can be inhibited by small molecule inhibitors that target this modification (Mathews and Hershey, 2015; Turpaev, 2018). In melanoma, EIF5A2 has been identified as a downstream effector of the PI3K/Akt signaling pathway. Overexpression of EIF5A2 markedly enhances the invasive capacity of melanoma cells and increases matrix metalloproteinase-2 (MMP-2) activity. Conversely, silencing of EIF5A2 produces the opposite effects, suggesting that EIF5A2 promotes melanoma cell invasion, at least in part, through upregulation of MMP-2 (Khosravi et al., 2014). Nevertheless, the underlying molecular regulatory mechanisms remain to be further elucidated. In contrast, direct functional studies investigating the role of EIF5A1 in melanoma are relatively limited. Notably, the antibody used in the immunofluorescence experiments in the present study specifically targets the EIF5A1 isoform, and our single-cell analyses focused on EIF5A1. However, the relationship between EIF5A1 expression changes observed at the transcriptomic level and the corresponding protein-level regulation and functional consequences remains unclear. Therefore, future studies should adopt an isoform-/gene-specific perspective to systematically elucidate the independent and potentially distinct biological functions and clinical significance of EIF5A1 and EIF5A2 in melanoma initiation, progression, and tumor immune regulation. Consistently, TCGA-SKCM bulk data suggest tumor upregulation and prognostic associations for both EIF5A1 (EIF5A) and EIF5A2 (Supplementary Figure S11).

This study integrates single-cell transcriptomic profiling with bulk prognostic modelling to characterize tumour cell functional dynamics and their influence on the immune microenvironment in melanoma. We observed that elevated EIF5A expression is correlated with poorer patient prognosis, suggesting its potential as an unfavourable prognostic marker and therapeutic target. Nonetheless, several limitations of this study warrant acknowledgment. The MRS was derived and evaluated primarily within TCGA-SKCM, with model performance assessed through internal resampling and a hold-out validation split. Although strict separation between training and testing sets was maintained throughout feature selection and model fitting, model selection among the 101 algorithm combinations was guided by internal cross-validation performance, which may introduce model selection bias; further validation in independent external cohorts with harmonized clinical annotations would therefore strengthen the generalizability of the signature, and these findings should currently be regarded as hypothesis-generating and a foundation for future confirmatory studies. Beyond the prognostic model, the single-cell analyses were based on a publicly available dataset with a relatively limited sample size, and the causal relationships implied by these observations remain to be formally established through *in vitro* or *in vivo* perturbation experiments, which we consider a priority direction for subsequent mechanistic work.

## 5 Conclusion

This study revealed the functional heterogeneity of melanoma cells and their dynamic crosstalk with the tumor microenvironment. Notably, three distinct subpopulations—Melanoma4, Melanoma6, and Melanoma9—were significantly associated with patient prognosis and exhibited extensive interactions with immune cells, particularly through the MIF (Macrophage Migration Inhibitory Factor) and MHC-I (Major Histocompatibility Complex class I) signaling pathways. By integrating machine learning approaches, we constructed a prognostic model based on melanoma cell-related signatures (MRS), which demonstrated consistent predictive performance across both training and validation cohorts, with a mean C-index of 0.675. Further analysis of key genes in the MRS identified EIF5A (Eukaryotic Initiation Factor 5A) as a plausible prognostic biomarker candidate and potential therapeutic target that warrants mechanistic validation in melanoma. Collectively, these findings provide a foundation for developing more precise and personalized treatment strategies for melanoma patients. Future studies with larger clinical cohorts and experimental validation are warranted to consolidate these conclusions and facilitate their clinical translation.

## Data Availability

The datasets presented in this study can be found in online repositories. The names of the repository/repositories and accession number(s) can be found in the article/Supplementary Material.
