# Supplementary material for: Single-cell sequencing reveals the functional heterogeneity of melanoma cells and their crosstalk with the tumor microenvironment
Source: Front Genet. 2026 Apr 2;17:1779213. doi: 10.3389/fgene.2026.1779213 (PMC13082755; doi:10.3389/fgene.2026.1779213)
Supplement: Supplementary file 1 [file DataSheet1.pdf]

Supplementary information

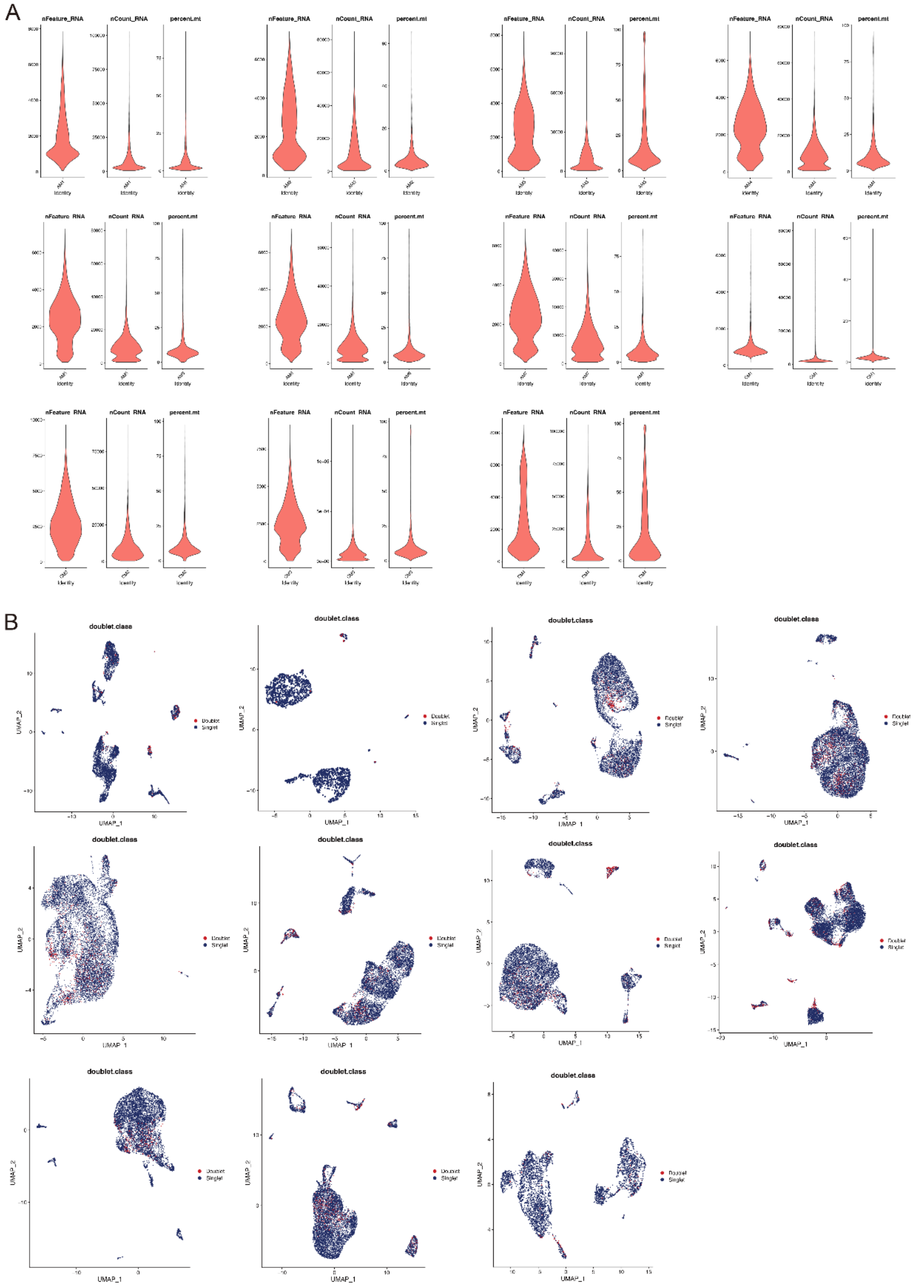

Figure S1: Quality control of scRNA-seq data from. (A) Displays the results of sample quality control, showcasing violin plots for nFeatures_RNA, nCount_RNA, and mitochondrial percentage, respectively. (B) Calculates the inferred doublet cells in each sample.

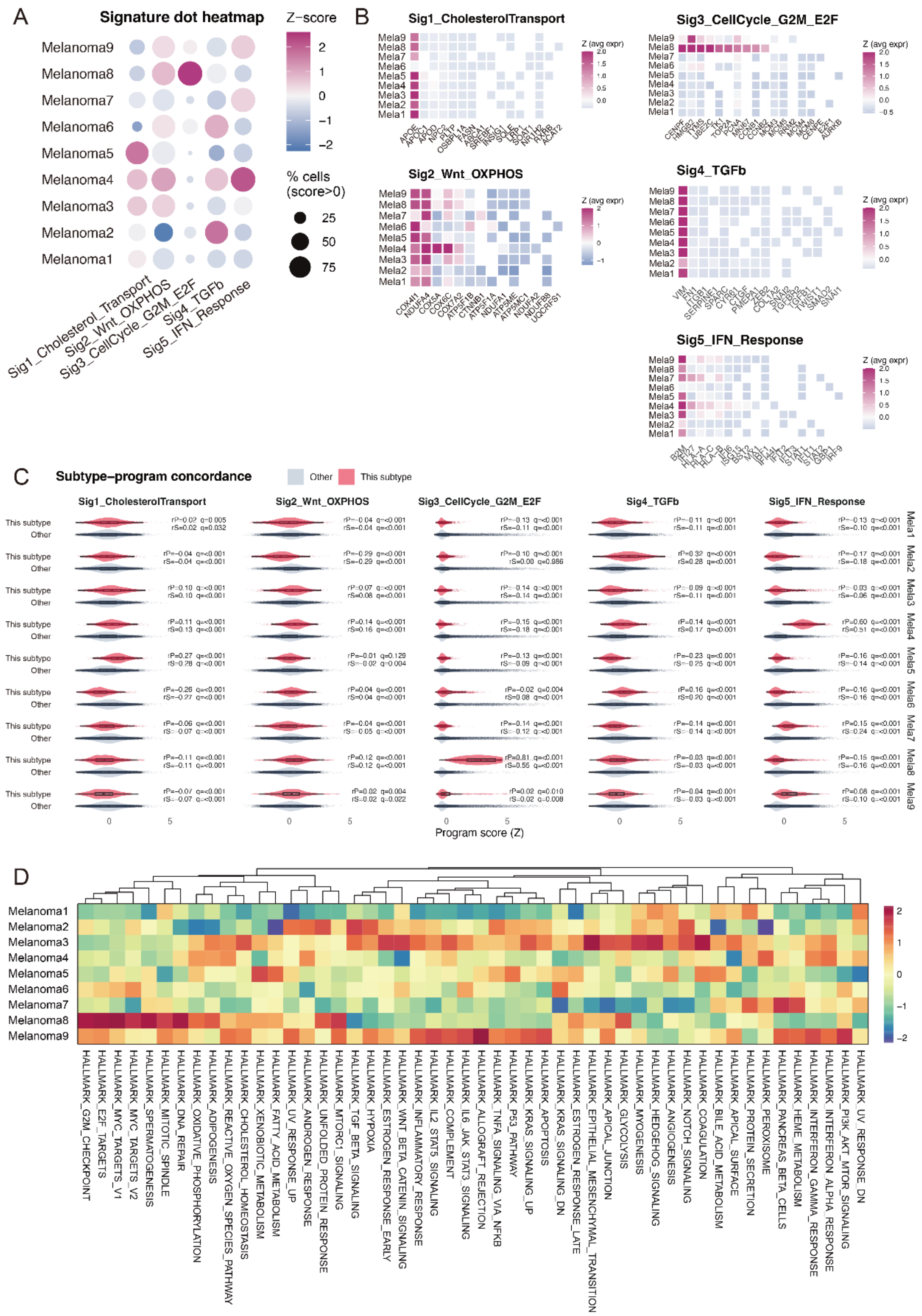

Figure S2. Mapping of melanoma malignant subpopulations (Mela1–Mela9) to the tumor-intrinsic programs defined by *Zhang et al.* (A) Signature dot heatmap showing signature activity across Mela1–Mela9. Signature scores were calculated at the single-cell level using Seurat AddModuleScore based on curated gene sets. Color indicates the z-scored mean signature score (computed within each signature across subpopulations), and dot size indicates the percentage of

cells with a positive score in each subpopulation. (B) Heatmaps of representative high-expression genes for each signature across subpopulations. (C) Subtype–signature concordance analysis comparing signature-score distributions between cells in a given subpopulation (“This subtype”) and cells from all other subpopulations (“Other”). Each row corresponds to one melanoma subpopulation and each column corresponds to one signature. Pearson (rP) and Spearman (rS) correlations between signature score and subpopulation membership are shown with Benjamini–Hochberg (BH) adjusted q-values. (D) Heatmap of MSigDB Hallmark pathway activities across malignant melanoma subpopulations (Mela1–Mela9), computed by GSVA on Seurat AverageExpression profiles. Values are row-scaled z-scores (hierarchical clustering of rows).

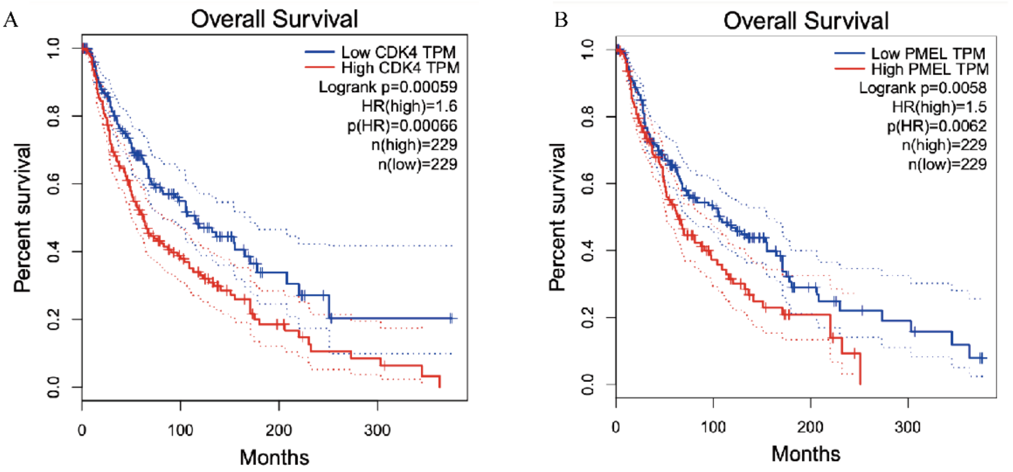

Figure S3: High expression of CDK4 and PMEL in the TCGA-SKCM dataset is linked to poorer patient prognosis.

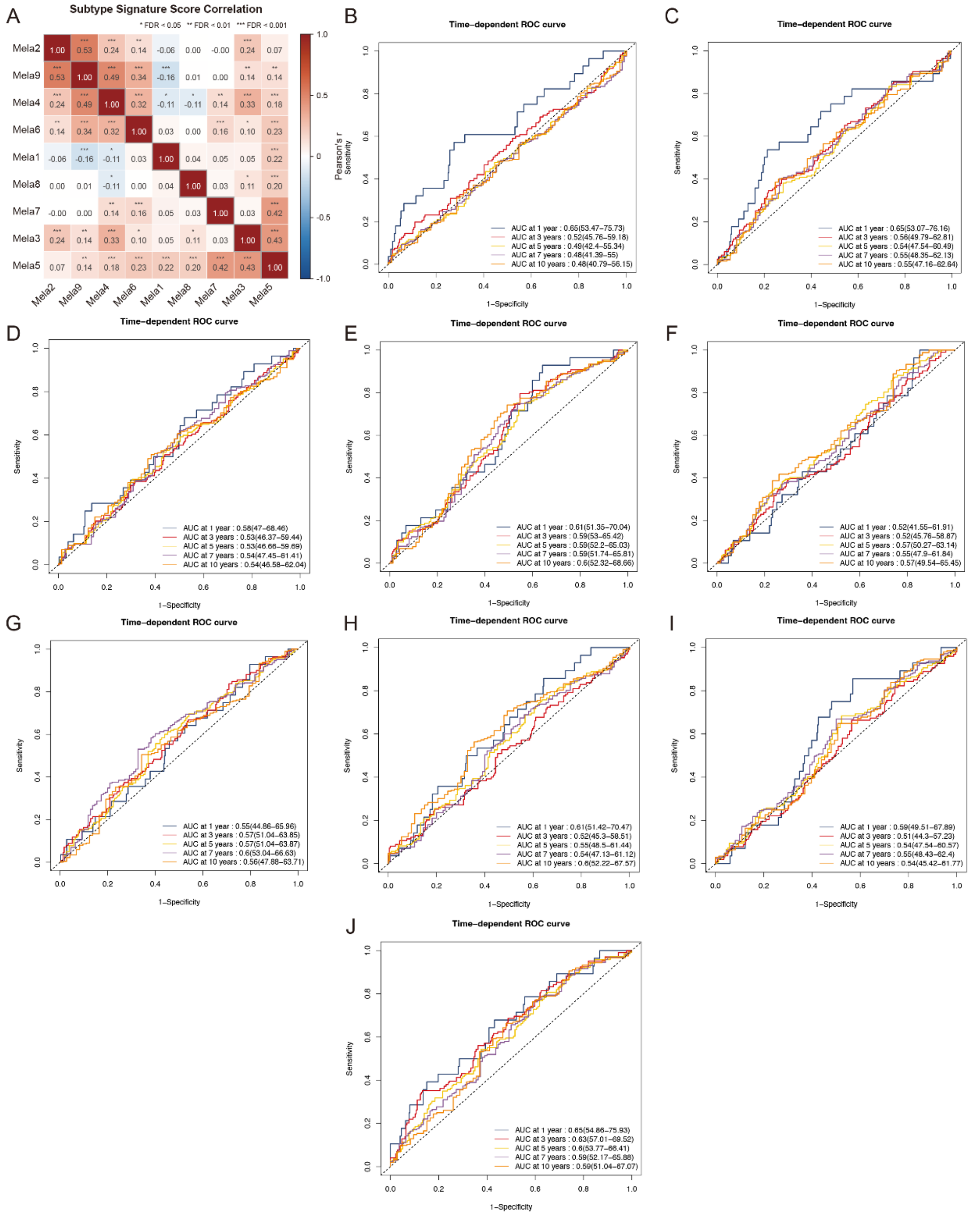

Figure S4: (A) Correlation heatmap of TCGA-SKCM melanoma subpopulation (Mela1–Mela9) signature scores, with Pearson's r shown in each cell and FDR-adjusted significance indicated by asterisks. (B-J) ROC curves displaying AUC at 1, 5, 7, and 10 years to assess the effectiveness of each melanoma subgroup in predicting patient overall survival

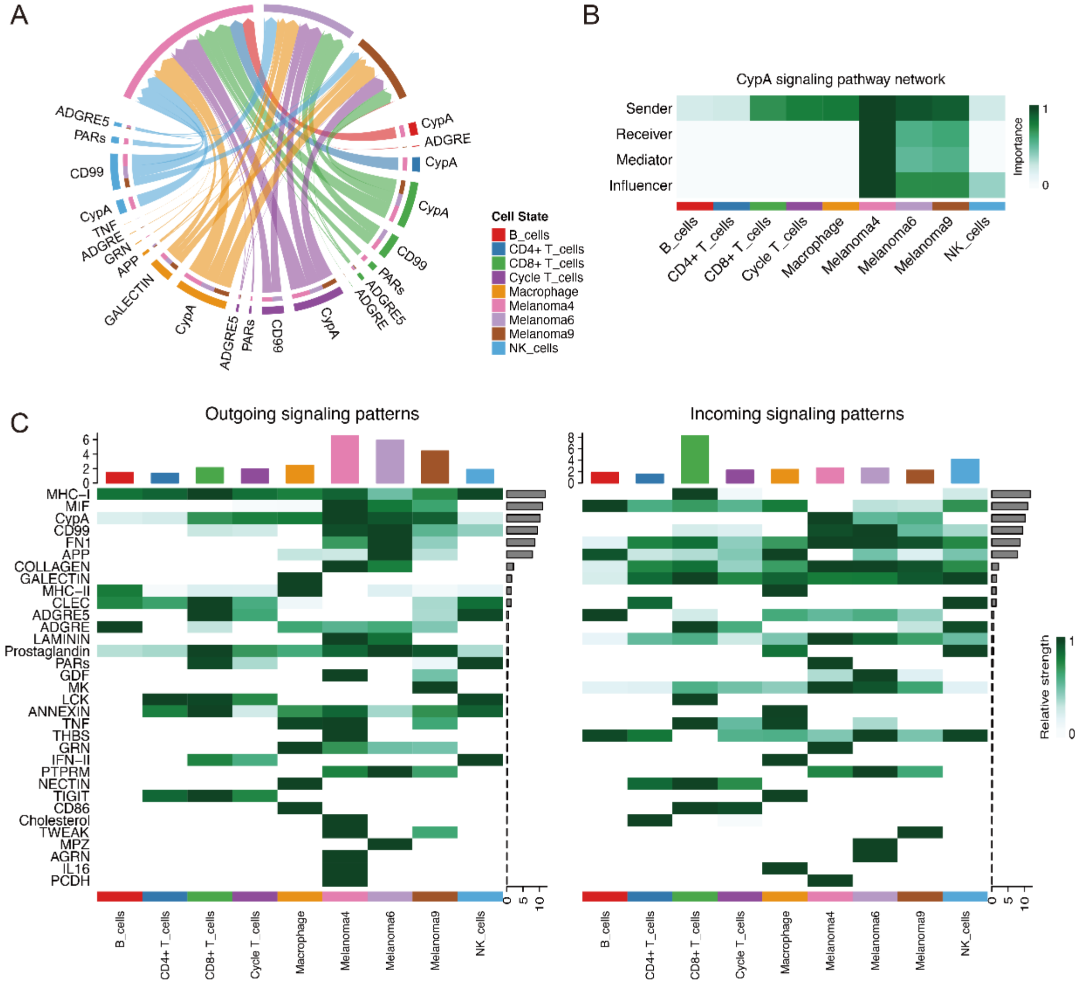

Figure S5: (A) Circle plot visualizing receptor-ligand interactions between melanoma subgroups and various immune cells. (B) Heatmap displaying the contribution of individual cell types to the CypA signaling pathway. (C) Heatmap illustrating the efferent or afferent contributions of all signals to different groups of immune cells.

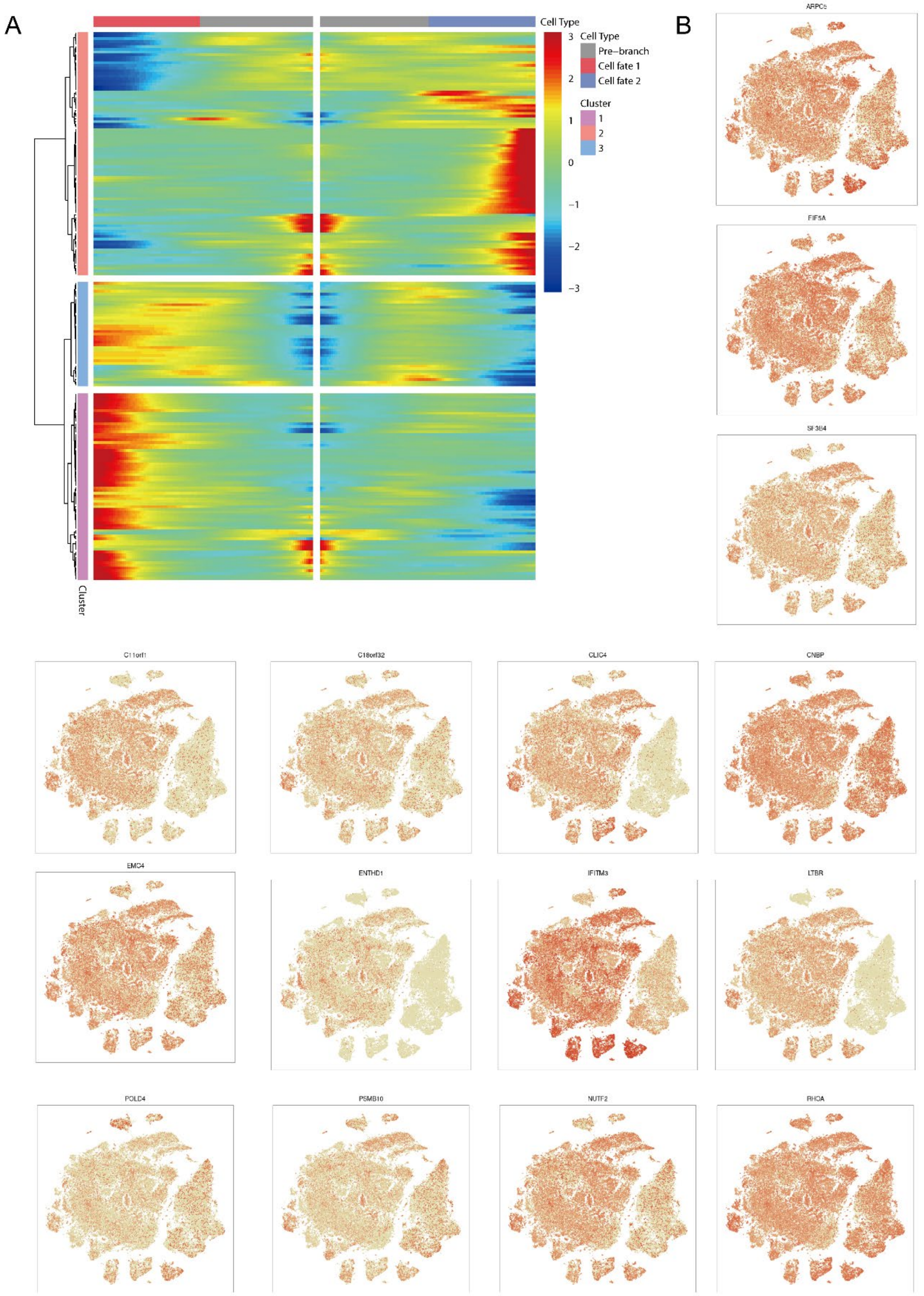

Figure S6: (A) Heatmap depicting the dynamic changes in the expression of the top 200 genes before and after branch node 1. (B) Expression of each gene constituting the Melanoma Resilience Score (MRS) in melanoma single-cell data.

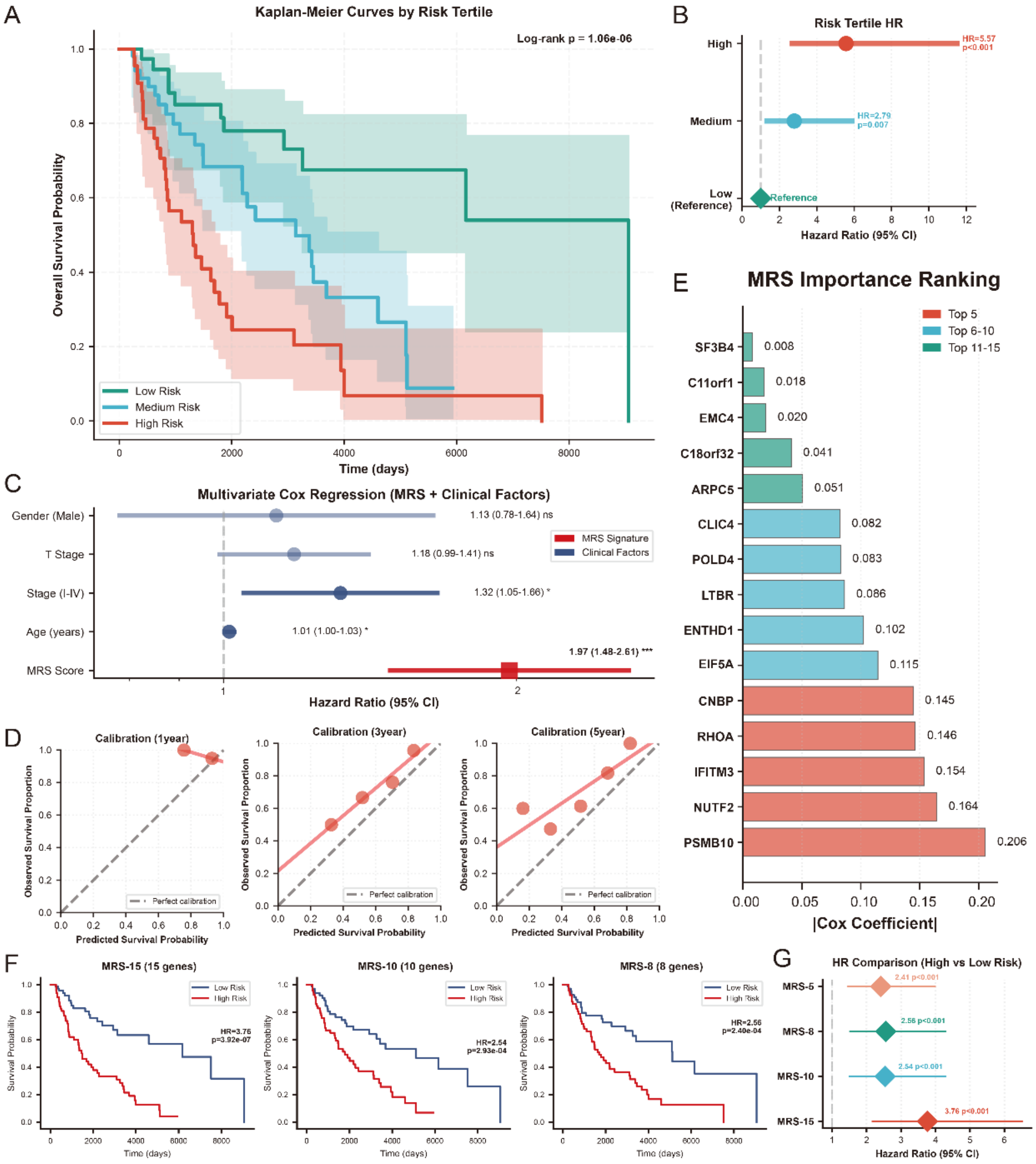

Figure S7. Prognostic performance, clinical independence, calibration, and ablation analyses of the 15-gene MRS. (A) Kaplan–Meier overall survival curves in the validation set stratified by MRS risk tertiles (Low/Medium/High). Shaded areas indicate 95% confidence intervals. The log-rank P value is shown. (B) Hazard ratios (HRs) for Medium and High tertiles versus the Low tertile (reference) estimated by Cox proportional hazards models in the validation set; points indicate HRs and horizontal lines indicate 95% CIs. (C) Multivariable Cox regression evaluating the independent prognostic value of MRS after adjustment for clinicopathologic covariates (age, sex, AJCC pathologic stage, and pathologic T stage). Horizontal bars denote 95% CIs and significance is annotated as ns, ∗ P<0.05, ∗∗ P<0.01, ∗∗∗ P<0.001. (D) Calibration plots for 1-, 3-, and 5-year survival in the validation set. Predicted survival probabilities from the Cox model are grouped into bins and compared with observed survival proportions; the dashed diagonal indicates perfect calibration. (E) Importance ranking of the 15 MRS genes based on the absolute value of Cox

regression coefficients |β| fitted in the training set; colors denote top-5, ranks 6–10, and ranks 11–15. (F) Kaplan–Meier curves in the validation set comparing High- versus Low-risk groups defined by the median risk score for different signature sizes (MRS-15, MRS-10, and MRS-8). (G) Summary forest plot comparing HRs (High vs Low) across signatures of different sizes in the validation set; points indicate HRs and horizontal lines indicate 95% CIs.

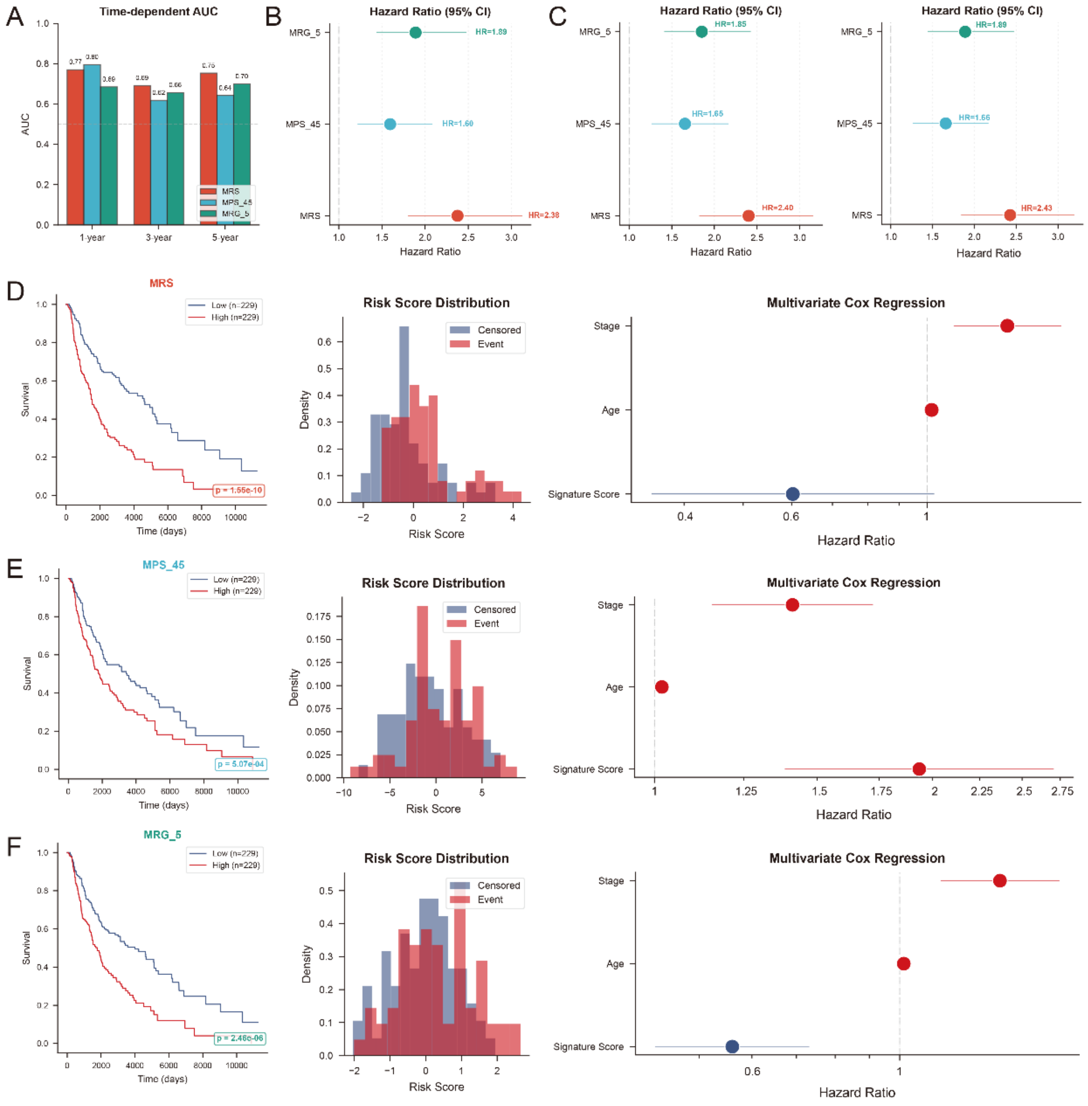

Figure S8. prognostic performance comparison of MRS with published melanoma signatures. (A) Time-dependent AUC at 1, 3, and 5 years for the 15-gene MRS and two published signatures (MPS_45 and MRG_5) in the TCGA-SKCM cohort. (B-C) Hazard ratios (HR) with 95% confidence intervals for the high- vs low-risk groups defined by each signature using median risk-score dichotomization. Panels show representative results from independent random train/validation splits. (D-F) Kaplan–Meier (KM) survival curves (left), risk-score distributions stratified by vital status (middle), and multivariable Cox regression forest plots (right) for MRS (D), MPS_45 (E), and MRG_5 (F). High- and low-risk groups were defined by the cohort median risk score. Multivariable Cox models included the signature score together with clinical covariates. Points indicate HRs and horizontal lines indicate 95% confidence intervals; the dashed vertical line marks HR = 1.

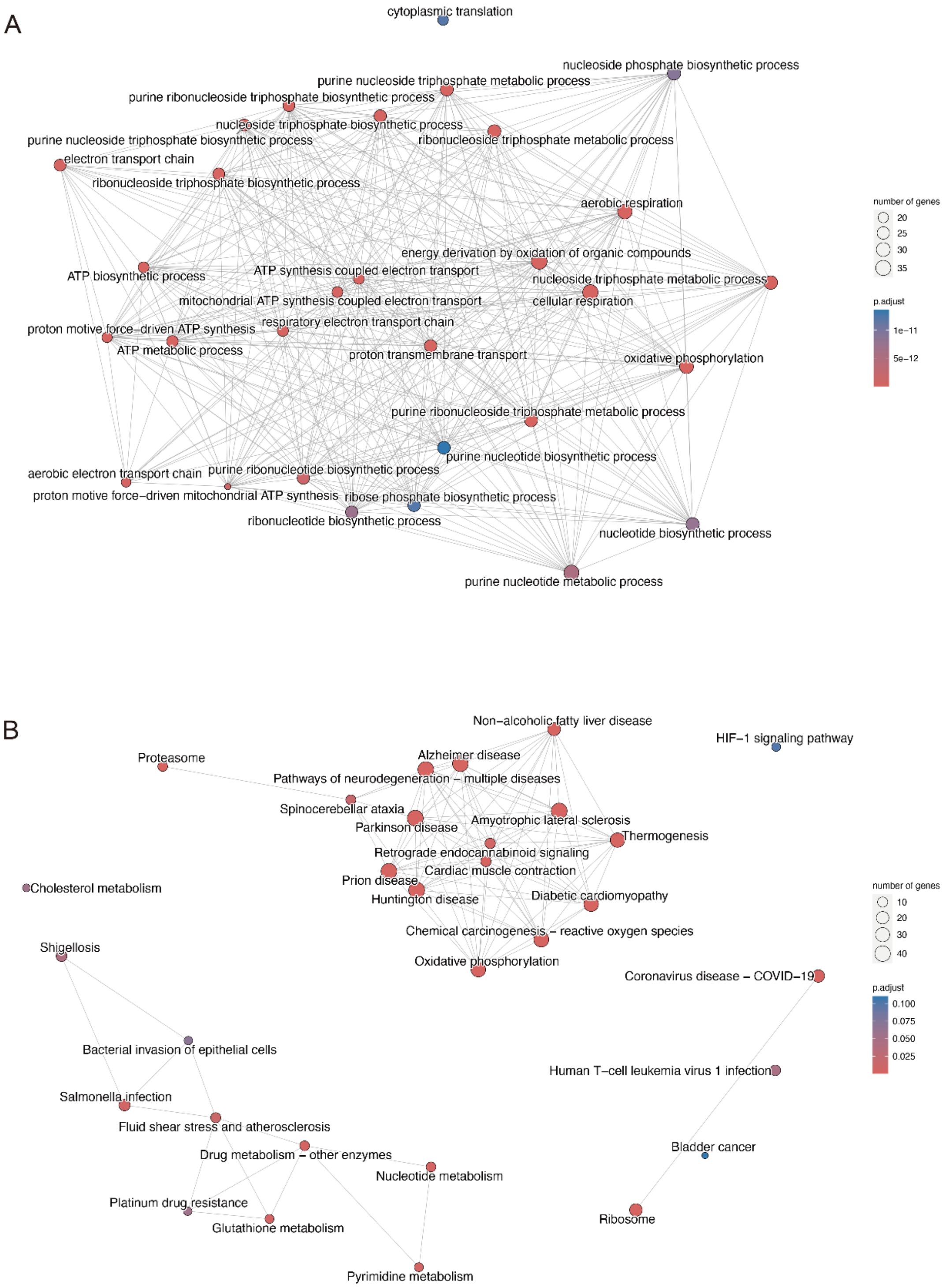

Figure S9: (A-B) Network diagrams of significant entries in functional enrichment analysis for differential genes in the EIF5A (+) and EIF5A (-) groups:(A) Gene Ontology (GO) entries. (B) Kyoto Encyclopedia of Genes and Genomes (KEGG) entries.

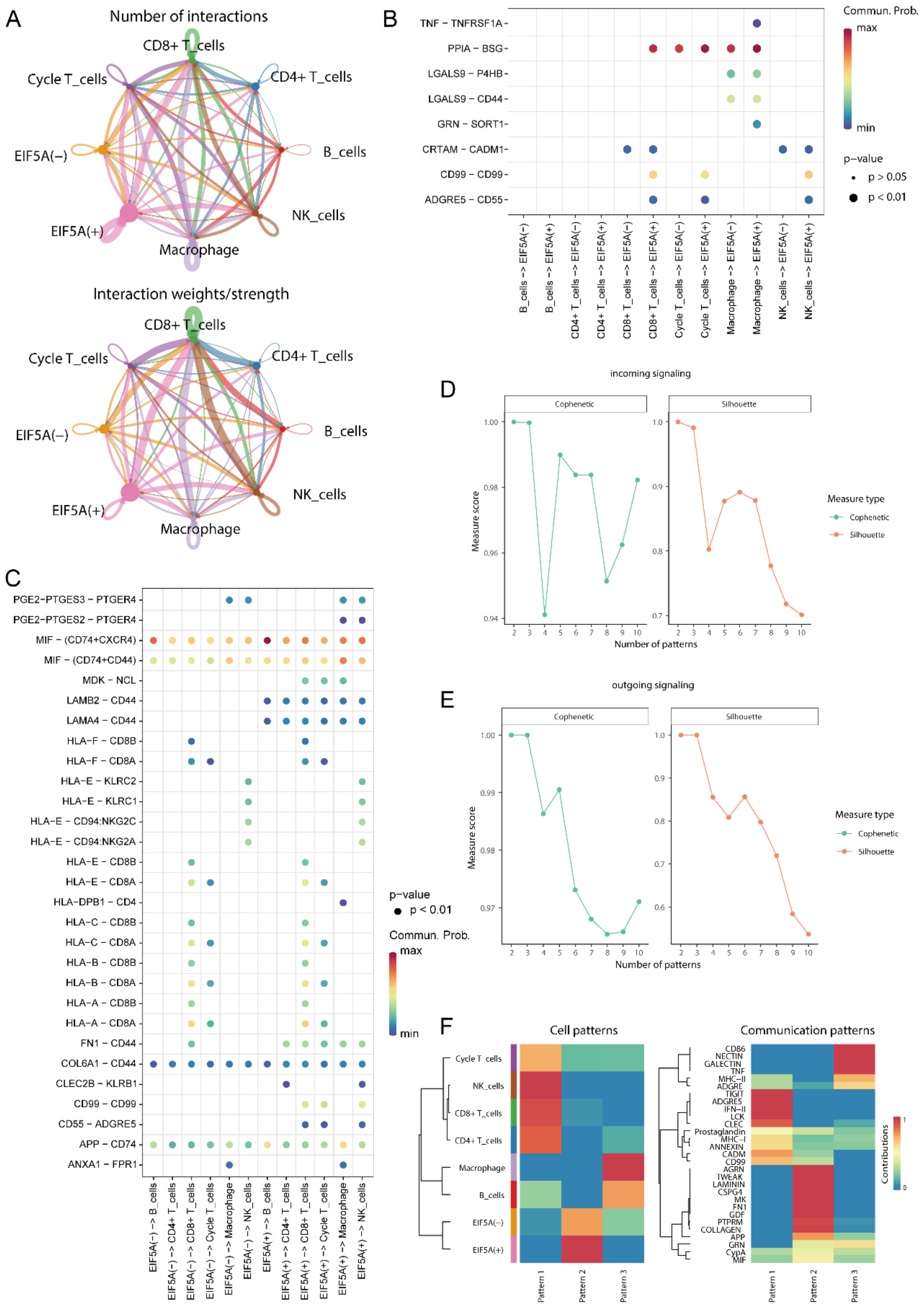

Figure S10: (A) Circle plot showing the number (top) and intensity (bottom) of interactions between EIF5A (+), EIF5A (-) cells, and immune cells. (B-C) Bubble plots illustrating overexpressed ligand-receptor interactions: (B) EIF5A (+) and EIF5A (-) melanoma cells as signal receivers. (C) Signal senders. The bubble size represents the P-value generated by the permutation test, and the color indicates the likelihood of interaction. (D-E) Number of: (D) Outgoing signals. (E) Incoming signals,

based on Cophenetic and Silhouette indexes. (F) Heatmap displaying the contribution of different signals to groups of cells.

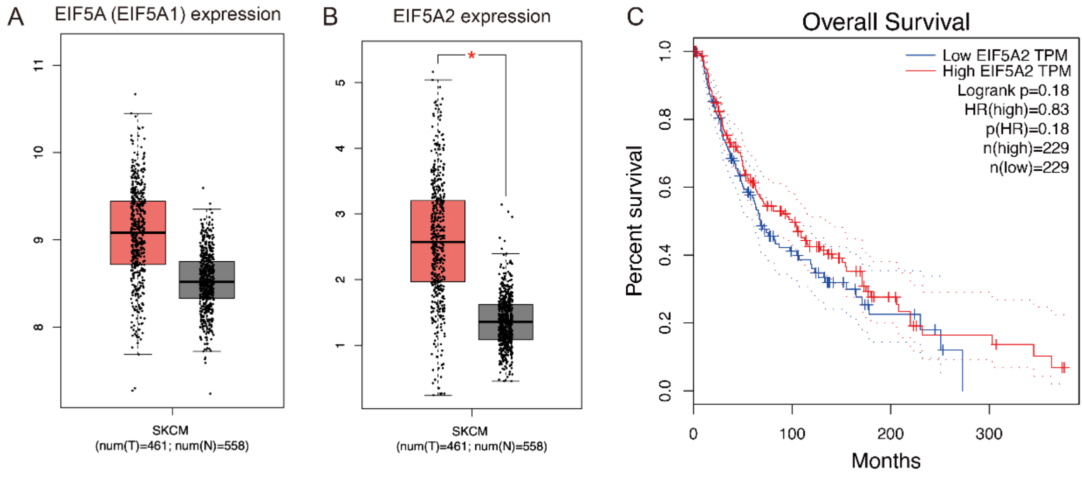

Figure S11. Isoform-specific EIF5A expression and survival association in TCGA-SKCM. (A–B) EIF5A1 and EIF5A2 expression in tumor versus normal samples. (C) Kaplan–Meier overall survival stratified by high versus low EIF5A2 expression.
